# Fibroblast Growth Factor-2 (FGF-2) Expression in Pterygia Using Cell Spot Arrays

**DOI:** 10.3390/vision6040058

**Published:** 2022-09-22

**Authors:** Stylianos Mastronikolis, Evangelos Tsiambas, Konstantinos Kagkelaris, Marina Pagkalou, Panagiotis Plotas, Sofianiki Mastronikoli, Dimitrios Roukas, Constantinos D. Georgakopoulos

**Affiliations:** 1 Department of Ophthalmology, Medical School, University of Patras, 26504 Patras, Greece; 2 Department of Cytology, 417 Veterans Army Hospital (NIMTS), 11521 Athens, Greece; 3 Department of Chemistry, University of Crete, 71500 Voutes, Greece; 4 Department of Primary Health Care, School of Health Rehabilitation Sciences, University of Patras, 26504 Patras, Greece; 5 Brighton and Sussex Medical School, University of Sussex, Brighton BN1 9PX, UK

**Keywords:** pterygia, FGF, growth factors, image analysis, cytology

## Abstract

Fibroblast growth factor (FGF) is a main regulator of cell differentiation, cell migration and angiogenesis in normal and abnormal conjunctiva epithelia, but specific mechanisms of its aberrant expression are yet to be investigated. In the present study, we investigated FGF-2 protein expression within several pterygia. Using a liquid-based cytology assay, we obtained cell specimens from pterygia and healthy tissues directly from patients. A combination of immunocytochemistry followed by digital image analysis showed significant overexpression of FGF-2 in all the examined pterygia. In 30/60 (50%) cases there were high levels of staining intensity, whereas in the remaining 30/60 (50%) cases there were moderate levels of expression. FGF-2 levels of the control group were significantly lower in comparison with the pterygia group. There was no significant correlation between FGF-2 levels and either sex or location of the pterygium. FGF-2 levels had a significant correlation with morphological characteristics of the pterygia. More specifically, FGF-2 levels were significantly higher in the pterygia with a fleshy morphology. Interestingly, recurrent lesions demonstrated high expression levels. An overexpression of FGF-2 has been observed frequently in pterygia, where it may play a crucial role in determining the lesion’s progression. FGF-2 upregulation correlates with the morphology of pterygia and its tendency to recur. Cell spot analysis based on liquid-based cytology is a simple, yet effective, method for detecting a broad spectrum of protein markers and could be useful in analyzing potential pterygia patient samples.

## 1. Introduction

Pterygium refers to an ocular surface non-neoplastic (although mimicking) lesion characterized by specific clinicopathological features. Histopathologically, it demonstrates abnormal proliferation of fibroblasts and vessels, as well as chronic inflammatory infiltration, leading to degenerative and hyperplastic conjunctival epithelia [1,2]. Significant clinical signs are corneal dellen, progressive irregular astigmatism, as well as possible vision loss. All clinical signs are mediated by the invasion of the cornea tissue from these modified tissues [3,4]. Concerning epidemiology, pterygium often occurs in adults, with an increase in incidence in certain geographic regions [5,6]. Main etiopathogenetic factors include chronic high exposure to ultraviolet (UV) radiation, viral infections, genetic susceptibility and prolonged inflammation [7]. Aberrant UV exposure results in DNA damage and plays a central role in the transformation of the epithelial microenvironment [8].

Among molecules that are implicated in pterygia development and progression, growth factors provide histogenesis and formation in epithelia and their deregulation leads to specific abnormalities. In particular, fibroblast growth factor (FGF) is a protein of major importance. The FGF family comprises some protein isoforms, including acidic and basic FGFs (1/2, respectively). The *FGF-2 gene* (gene locus: *4q28.1*) encodes for a protein that acts as an important regulator of cell differentiation, cell migration and angiogenesis in normal and abnormal epithelia [9]. Additionally, the wound healing process, nervous and limb system development and functional maturation, combined with specific autocrine and paracrine effects, are FGF-mediated specific activities [10,11]. The present study analyzed the levels of FGF-2 in pterygia and normal conjunctiva epithelial cells by implementing the cell spot array technique on cytological slides combined with a digital image analysis algorithm to objectively measure the corresponding protein levels. 

## 2. Materials and Methods

### 2.1. Study Design and Patients

Sixty (*n* = 60) Caucasian individuals in Greece suffering from pterygia lesions participated in the present study. Their ages ranged from 37 to 82 years with a mean age of 63.2 years. They consisted of forty females and twenty males. According to The Declaration of Helsinki guidelines, all patients were informed of the present study and gave consent to have their samples used for research purposes. All data presented were collected by researchers who are also treating physicians. This study was directed by the Department of Ophthalmology and approved by the Research Ethics Committee of the Medical School, University of Patras, Rio-Patra, Greece (Decision reference number: 310/06.07.2017).

### 2.2. Cell Substrates

**Figure 1:**
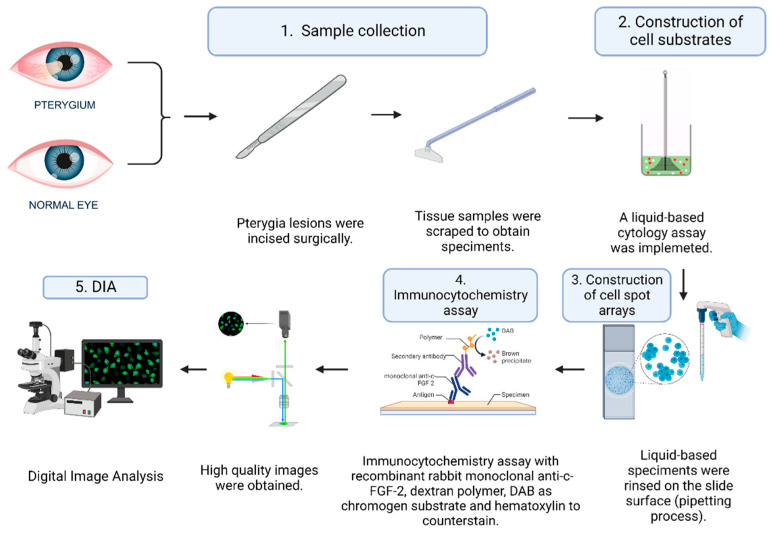
The specimen collection and analysis procedure. FGF: Fibroblastic Growth Factor; DAB: 3-3, diaminobenzidine tetrahydrochloride.

For our study, sixty (*n* = 60) conjunctiva epithelia were scraped in a smooth manner to obtain cell specimens. All cell specimens were collected and fixed using a liquid-based cytology assay (Cell Solutions, Menarini, Florence, Italy). All patients examined were questioned regarding potential HPV infection diagnosed via PCR in their medical history and declared no infection or other comorbidity. The same procedure was carried out on normal conjunctival epithelia (*n* = 20; control group). In 26 of the examined cases, pterygium was recognized in only one eye. Thirty-four (*n* = 34) cases were characterized by bilateral lesion. All control group (normal appearing) epithelia were obtained from the non-pterygium areas of the fellow eyes. Histopathological features were confirmed via biopsy. In order to define their subtypes, two MD experts (ophthalmologists) categorized pterygia according to their clinical features and macroscopic morphology. The final agreement was 100%, although for one case there was a slightly different opinion. Concerning the last case, the two pathologists reached a final agreement based on the area and depth of the lesion. All patients underwent the bare sclera technique for pterygium excision, performed by the same surgeon (Figure 1). 

### 2.3. Construction of the Slide for the Cell Spot Array

**Figure 2:**
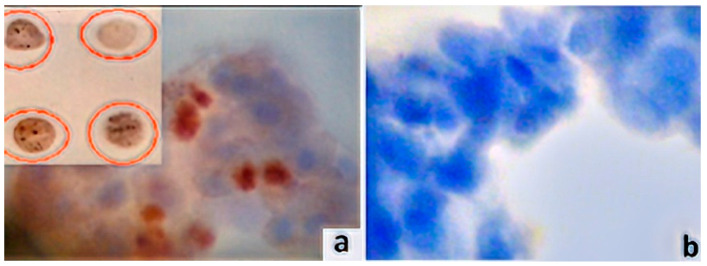
FGF-2 protein expression patterns in pterygia fibro-endothelial cells (original magnification 400×). (**a**) FGF-2 high expression in a cell spot. Note dark brown nuclear/perinuclear staining pattern. Inset image: cell spot arrays. Spots were obtained by scraping liquid-based cytological specimens (3-3, diaminobenzidine tetrahydrochloride (DAB) chromogen). (**b**) FGF-2 negative stain. Note absence of the marker’s expression (control); counterstained with hematoxylin.

Ten slides (BestPrep/Cell Solutions, Menarini, Florence, Italy) were constructed. Each contained eight cell spots. Equal amounts of each liquid-based specimen were rinsed on the slide surface (spot diameter: ~0.5 cm); a cell spot array on this same slide was the result of implementing a pipetting process by an expert cytotechnologist. A liquid-based cytology assay is an alternative to conventional cytology method that uses vials with an alcohol solution for the fixation of collected cell samples. On each slide, four spots were arranged in two columns. The microscopical examination of all (*n* = 80) examined pterygia and control cases revealed a single spot for each case (confirming the suitability of the specimens) (Figure 2a). 

### 2.4. Immunocytochemistry Assays (ICC) and Antibodies

Ready-to-use recombinant rabbit monoclonal anti-c-FGF-2 (clone EP1735IgG, Abcam, Waltham, MA, USA) following a dilution of 1:1000 with Tris-buffered saline (TBS) and 1% bovine serum albumin (BSA) addressed the respective cell spot array slides. EnVision protocol (DAKO, Glostrup, Denmark) was applied to perform ICC for the marker, implementing an automated IHC staining system (I 6000-Biogenex, Frentmont, CA, USA). ICC’s current method uses a water-soluble dextran polymer, which prevents the endogenous biotin reaction, which causes background in stained slides. In brief, a 30-min incubation with the primary antibody at room temperature was followed by a 30-min incubation with the polymer. As a chromogen substrate, 3-3, diaminobenzidine tetrahydrochloride (DAB) was used to visualize the antigen–antibody reaction. In the final step, after slight counterstaining with hematoxylin for 30 s, the slides were dried and mounted (Figure 1). The primary antibodies were omitted from the opposing control slides. Based on the manufacturer’s instructions, the predominantly nuclear (but also perinuclear/cytoplasmic) staining pattern was deemed as an acceptable indicator of the protein’s specificity (Figure 2b). Breast carcinoma tissue sections demonstrating FGF-2 expression were used as the positive marker.

### 2.5. Digital Image Analysis (DIA)

**Figure 3:**
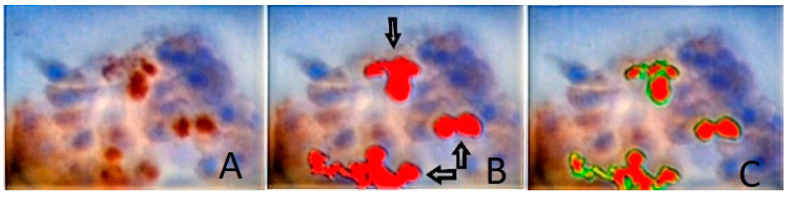
(**A**) Digital image analysis of a pterygia cell spot-stained using FGF-2 antibody (original magnification ×400). (**B**) Different expression values are visible in reddish areas (black arrows). (**C**) Green lines surround specific areas of interest (range: 0–255 grey scale immunostaining intensity levels).

Using stained pterygia and normal standard conjunctiva cell substrates, FGF-2 protein levels of expression were quantitatively evaluated through densitometry evaluation. The DIA was performed using a semiautomated system (hardware: Microscope CX-31, Olympus, Melville, NY, USA, digital camera, Sony, Tokyo, Japan; Windows XP/NIS-Elements Software AR v3.0, Nikon Corp, Tokyo, Japan). A digital database was used to store snapshots of the identified areas of interest per tissue section (5 optical fields at 400 magnification). Based on the manufacturer’s data sheet for monoclonal mouse anti-c-FGF-2, measurements were performed using a specific macro (nuclear/perinuclear staining pattern). Using an algorithm, normal (control) spot measurements were compared to the corresponding values in pterygia spots. For the red-green-blue (RGB) analysis, a wide range of continuous grayscale values (0–255) was available (Figure 3). The corresponding areas of interest were selected and demarcated manually during the image analysis procedure.

In the next step, a medley of RGB frequencies was utilized to compute the mean of all marked pixels based on an adjustable algorithm that employs grayscale LUTs. Therefore, the blue channel’s nuclear intensity in the immunostained (brown) areas was initially converted to a grayscale pattern. A combined RGB frequency analysis in cases of negative cytoplasmic staining demonstrated values that approached elevated white levels (up to 255). As the intensity of immunostaining decreased to 0, the marker was increasingly overexpressed, whereas as the immunostaining intensity increased to 255, its expression diminished. 

### 2.6. Statistical Analysis

Statistical analysis was performed by applying SPSS statistical package version 28.0 (Statistical Package for the Social Sciences, version 28.0, SSPS Inc., Chicago, IL, USA). Assessment of associations between variables was carried out using the Pearson Chi-Square (χ^2^) test and Fisher’s exact. The χ^2^ analysis was used to compare FGF-2 levels (low or moderate/high) between the control and pterygia groups. Logistic regression analysis was conducted to examine the relationships between FGF levels in the pterygia and sex, location and morphology of the pterygia, and the presence of (or lack of) recurrent lesions. The Spearman rank correlation test was used to analyze variables that had significant chi^2^ associations. We considered two-tailed *p*-values of less than 0.05 as statistically significant. 

## 3. Results

The aforementioned DIA protein analysis revealed that all examined cases exhibited varying levels of FGF-2 immunostaining. Nuclear/perinuclear staining immunoreactivity was detected in small fragments of fibrous tissue as well as epithelial and endothelial cells. The marker was overexpressed in all of the pterygia examined. There were high staining intensity levels in 30/60 (50%) samples, whereas the remaining 30/60 (50%) showed moderate expression. The pterygia group demonstrated significantly elevated FGF-2 levels in comparison to the control group (*p =* 0.001). No significant correlation was found between FGF levels and sex (*p =* 0.469) or the location [12] of the pterygia (*p =* 0.341). FGF-2 levels were significantly related to the morphology [12] of the pterygia (*p =* 0.007). More specifically, levels were significantly higher in pterygia with a fleshy morphology. Interestingly (although without statistical significance), recurrent pterygia revealed the highest levels of FGF-2 expression. Table 1 summarizes ICC results and corresponding *p*-values.

## 4. Discussion

Growth factors and their corresponding transmembrane receptors are involved in crucial signaling transduction pathways [13]. In particular, FGFs/FGFRs complexes play key roles in regulating cell and tissue functions involving differentiation, migration, proliferation, survival, cellular lineage commitment and apoptosis [14]. In fact, development, metabolism and tissue homeostasis are mediated by two FGF main types, FGF-1 and FGF-2 (acidic and basic, respectively) and their variations (isoforms) [15,16]. These molecules also strongly interact with cell adhesion factors, such as cadherins and integrins, modifying cell and cytoskeleton architecture [17]. Additionally, they are implicated in epithelial–mesenchymal transition phenomenon [18]. FGF/FGFR signaling dysregulation is partially responsible for the progression of neoplastic diseases, but not diseases such as epithelial malignancies (carcinomas), chronic kidney disease (CKD) or even insulin resistance [19,20,21]. In their inherent form, a variety of activating FGFR germline mutations have been already detected [22]. FGF/FGFR deregulation negatively affects epithelial neoplastic transformation in ocular diseases and tumors [23]. Uveal melanoma and retinoblastoma are characterized by a severe overexpression of the FGF/FGFR system, especially of the FGF-2 molecule [24]. Neoplastic (neo) angiogenesis plays a critical role in the progression of these malignancies; this mechanism is also observed in pterygia [25,26,27,28]. 

The present study investigates the role of FGF-2 aberrant expression in several cases of pterygia by applying a unique cell-based technique. As a component of angiogenesis, wound healing and various endocrine signaling pathways, FGF-2 plays an important role in these processes. An increase in bFGF expression was observed in epithelium, mast cells and blood vessels of pterygium. FGF-2 mRNA levels were found to be higher in pterygium and normal conjunctiva. It has been demonstrated that FGF-2 can induce inflammation and angiogenesis in pterygium by stimulating the expression of cyclooxygenase-2 (COX-2) [29]. We used a liquid-based cytology platform to co-examine FGF-2 protein expression levels of many cases on the same slide. According to our objective and accurate results, mediated by a digital image analysis assay, we reported moderate and high expression rates in these cases, whereas the control group (normal epithelia) demonstrated low to very low FGF-2 expression levels. A significantly higher expression was observed in pterygia with a fleshy morphology. Interestingly, the recurrent pterygia demonstrated the highest levels of FGF-2 expression. A similar study using cell cultures (conjunctival fibroblasts) reported increased immunoreactivity of FGF in recurrent (rather than in primary) pterygium fibroblasts, which is evidence of a specific stromal mechanism activation [30]. A number of fibroangiogenic growth factors were also overexpressed, including platelet-derived growth factor (PDGF), tumor necrosis factor alpha (TNF-alpha) and transforming growth factor beta (TGF-beta). Additionally, coactivation of growth factors (FGF-2/EGF/TGF) and inflammatory proteins, such as interleukins (IL-1b), necrosis factors (TNFa) and matrix metalloproteinases (MMPs) negatively affect the normal ocular surface inducing the onset and progression of pterygia [31,32]. Concerning cell types that demonstrate elevated FGF-2 protein expression in pterygia, a study group detected high levels using immunocytochemistry in endothelial cells of vessels, epithelial cells, fibroblasts, basement membranes and combined with inflammatory infiltration [33]. Furthermore, co-overexpression of FGF-2 and TGF-a/b growth factors is strong evidence of their critical interaction in the development and progression of pterygia [34,35]. Their impact on pterygia biological behavior is enhanced not only by the aberrant expression of hypoxia-inducible factor (HIF-1a/b), but also by vascular endothelial growth factor (VEGF) [36,37,38].

## 5. Conclusions

In conclusion, pterygia is characterized by a high level of FGF-2 expression that possibly plays a crucial role in the development and progression of the lesion. FGF-2 activation correlates with the morphology of pterygia and its tendency to recur. Concerning novel and sophisticated techniques in modern research regarding ocular diseases, cell spot analysis utilizing liquid-based cytology is an innovative, easy-to-use technique for analyzing a broad spectrum of protein markers in the ophthalmology field and in pterygia diagnosis.

## Figures and Tables

**Table 1:** FGF-2 expression results.

Clinicopathological Parameters		FGF-2 Expression		*p*-Value
	*n* (%)	H and M	L	
Pterygia Controls	*n* = 60 *n* = 20	60/60 (100%) 3/20 (15%)	0/60 (0%) 17/20 (85%)	***0.001***
**Gender**				0.469
Male	20/60 (33%)	20/20 (100%)	0/20 (0%)	
Female	40/60 (67%)	40/40 (100%)	0/40 (0%)	
**Anatomic location**				0.341
Central	32/60 (53%)	32/32 (100%)	0/32 (0%)	
Peripheral	28/60 (47%)	28/28 (100%)	0/28 (0%)	
**Morphology type**				***0.007***
Flat	36/60 (60%)	36/36 (100%)	0/36 (0%)	
Fleshy	24/60 (40%)	24/24 (100%)	0/24 (0%)	
**Recurrence status**				0.999
Relapse	8/60 (16%)	8/8 (100%)	0/8 (0%)	
Non-relapse	52/60 (84%)	52/52 (100%)	0/52 (0%)	

H and M: High and moderate overexpression (staining intensity values ≤ 143 (spectrum between 89 and 142); L: Low expression (staining intensity values ≥ 151 (spectrum between 151 and 179).

## Data Availability

Patient data are protected under HIPAA and are unavailable.
